# Media Exposure and Anxiety during COVID-19: The Mediation Effect of Media Vicarious Traumatization

**DOI:** 10.3390/ijerph17134720

**Published:** 2020-06-30

**Authors:** Cong Liu, Yi Liu

**Affiliations:** School of Media and Communication, Shanghai Jiao Tong University, Shanghai 200240, China; lcong26@sjtu.edu.cn

**Keywords:** COVID-19, media vicarious traumatization, anxiety, media exposure, official media, commercial media, Wuhan

## Abstract

The rapid spread and high death rates of the COVID-19 pandemic resulted in massive panic and anxiety all over the world. People rely heavily on media for information-seeking during the period of social isolation. This study aimed to explore the relationship between media exposure and anxiety, and highlighted the underlying mechanisms mediated by the media vicarious traumatization effect. A total of 1118 Chinese citizens participated in the online survey, who were from 30 provinces in mainland China. Results showed that all four types of media (official media, commercial media, social media, and overseas media) cause vicarious traumatization to their audiences to different degrees. It was also found that the impact of media exposure on anxiety was mediated by media vicarious traumatization: there were full mediation effects for commercial media exposure and overseas media exposure, while there were indirect-only mediation effects for official media exposure and social media exposure. Audiences staying in cities with a relatively severe pandemic were more susceptible to the vicarious traumatization caused by commercial media compared to those staying in Hubei. This study expanded the concept and application of vicarious traumatization to the mediated context, and the findings provided insightful advice to media practitioners in the face of major crisis.

## 1. Introduction

**Figure 1:**
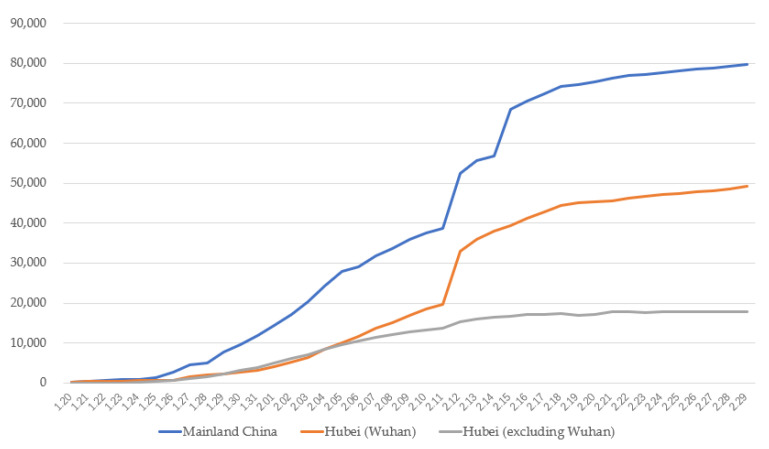
Number of confirmed cases in mainland China and Hubei Province from 20 January.

At the end of 2019, the COVID-19 virus broke out in Wuhan, the capital of Hubei Province, China [1]. In order to deal with the pandemic crisis, Wuhan was closed, and the traffic stopped from January 23, 2020 [2]. Chinese citizens all over the country were required to isolate themselves at home, wear face masks when going out, have their body temperature measured, and be tracked when entering and leaving public places with a real-name QR (Quick Response) code. The period from late January to February was the most drastic outbreak stage of COVID-19 in mainland China, especially in the Hubei province. Within two months, the number of confirmed cases reached 79,824 in mainland China by the end of February [3]. The trends are depicted in Figure 1. The statistics shown in Figure 1 were provided by the National Health Commission of P.R. China as well as the Health Commission of Hubei Province.

On 30 March 2020, WHO (World Health Organization) declared COVID-19 a pandemic [4]. The U.S. imposed a travel ban on Europe from March 1. By the end of March, more than 60 countries and regions in the world have successively declared a state of emergency, and some countries or regions have taken measures such as state closure and city closure. By 18 June 2020, the virus had spread to more than 200 countries and regions around the world, with 8,061,550 confirmed cases and 440,290 deaths [5].

### 1.1. Crisis Event, Anxiety, and Media Dependence

The outbreak of COVID-19 can be classified as a typical crisis event, which is defined as events that are specific and surprising, creating high levels of uncertainty and the perception of a severe threat [6]. In facing the pandemic, people showed a great degree of panic and anxiety due to the rapid spread of the pandemic, the rapidly increasing number of confirmed cases and deaths, the global shortage of protection resources, and the collapse of medical resources. A survey showed that 79.3% of Chinese citizens showed anxiety in their reaction to the coronavirus and more than 30% showed fear and panic, while very few expressed positive emotions [7]. The pandemic also had a profound impact on the lives of Americans. A survey conducted by Pew research showed that 90% of US citizens said their life had changed as a result of the COVID-19 outbreak [8]. At the same time, people in social isolation have had more media contact. Media reports deliver large numbers of pieces of pandemic-related information. During March 16–22, the viewing of the big four broadcast networks in the U.S. increased nearly 19% versus the same week in 2019, the viewing of cable news networks increased 73% versus the same week in 2019; the number of weekly visitors to U.S. news websites reached 630 million, 68% higher than that of February 17–23 [9].

According to media dependency theory [10], during a severe social disruption, there is an unusually high need for information and sense-making by individuals and the mass media are generally perceived to best satisfy these needs [11]. Specifically, the public relied heavily on the media to obtain information regarding operationalized guidance to the public, on the response of organizations [12,13], and also on an exchange of views with others [14]. One of the reasons why people usually need more information in the context of crisis events is to reduce the anxiety caused by uncertainty in the crisis event [15,16,17,18]. Research showed that in the H1N1 epidemic, uncertainty and uncontrollable feelings were positively related to stress and anxiety [19]. Therefore, people actively engage in information-seeking from a variety of sources to reduce uncertainty in a crisis event so as to ease their anxious feelings [16,20].

### 1.2. Media Exposure and Vicarious Traumatization

In a time of highly developed media technologies, the information available on the COVID-19 outbreak was far beyond the demand for information; the media’s effect on uncertainty-reduction in mitigating anxiety needs to be revisited. In contrast, evidence suggests that repeated media exposure is more likely to produce anxiety through the effect of vicarious traumatization. 

The original concept of vicarious traumatization was developed in the professional context, which describes the changes that occurs in trauma workers such as nurses and doctors, mental health staff, and police officers, as a result of working with trauma survivors [21,22,23,24,25,26]. Narratives of overwhelming horror and pain by the trauma survivors was identified as one of the key mechanisms in the development of vicarious trauma for the trauma workers [22]. With the development of media technology, audiences are also exposed to overwhelming reports and vivid visual materials about trauma survivors’ experiences vicariously through different kinds of media sources. 

Many studies have found that media exposure during critical public events may further cause psychological trauma and anxiety, indicating that the media’s vicarious traumatization effect may play an important role. After the 9/11 attack, people exposed to more television images of people falling or jumping to their death reported a higher posttraumatic stress disorder (PTSD) [27,28]. Thompson and colleagues also argued that media exposure to mass violent events can fuel a cycle of distress [29]. In the case of the 1995 Oklahoma City bombing, children who watched extensive television coverage of the crisis had more post-traumatic stress symptoms in both short- and long-term assessments. During the outbreak of Ebola in 2004, the media were more inclined to report the negative content related to Ebola, including anxiety, depression, and impairment of physical function [30,31,32]. As a result, individuals consuming more trauma-related reports were more anxious about Ebola infection [33,34]. An increased amount of media consumption on the 9/11 attacks was also found to cause higher levels of psychological distress [35]. After the Boston Marathon explosion in 2013 and the pulse nightclub massacre in Orlando in 2016, a three-year national survey from 4165 samples in the U.S. showed a positive link between contact with trauma-related media information and anxiety [36]. 

### 1.3. Factors Moderating the Impact of Vicarious Traumatization

Factors moderating the development of vicarious trauma have been explored in previous studies, which include the amount or length of time of exposure to trauma [37,38] and personal trauma experience [39,40,41]. Evidence also revealed that geographic location causes differences in the link between media exposure and psychological distress. Individuals in close proximity to the site of the 9/11 attacks reported more fear than those who were further away [42]. During the outbreak of COVID-19 in China from late January 2020, people in different areas were exposed to various degrees of direct trauma as well as vicarious trauma via media. In specific, people staying in Wuhan and other regions of Hubei province had experienced the top severity of pandemic and direct trauma, while people staying in cities such as Beijing, Shanghai, and Guangzhou had experienced comparatively severe pandemic as well as vicarious trauma via media. Therefore, the media’s impact on vicarious traumatization can be different across regions in the context of COVID-19.

### 1.4. The Current Study

In sum, despite evidence found supporting the link between media exposure and psychological distress such as anxiety, little is known about any underlying mechanisms, especially on how media vicarious traumatization may mediate this effect. Moreover, it is also unclear whether other factors relating to media sources and geographic location will make any difference. 

**Figure 2:**
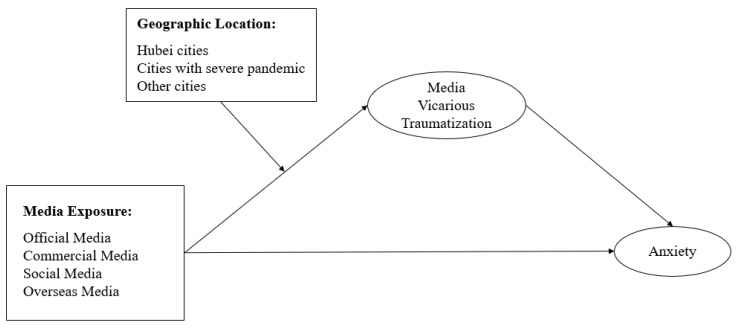
Conceptual framework.

The current study will investigate Chinese citizen’s media use and psychological status, particularly during the most drastic development of the COVID-19 pandemic in China from late January to February 2020. It aims to examine three research questions: (a) whether the link between media exposure and anxiety is mediated by media vicarious traumatization, (b) what the differences across types of media sources are, and (c) how geographic location will moderate the effect of media exposure on vicarious traumatization. The conceptual framework of the study is shown in Figure 2. 

Considering the different media typologies and the impact of media on Chinese audiences during the COVID-19 pandemic, media sources will be classified into government official media, commercial media, social media, and overseas media. The government official media, which represent the spokesmen of the government, such as CCTV, People’s Daily, and Hubei Daily, can be accessed on both traditional media (e.g., via TV channels and newspapers) and new media (e.g., official accounts on social media platforms). Commercial media, such as The Paper, Sanlian Life Week, and Caixin, are mostly accessed on new media platforms (e.g., applications developed by the media, accounts on social media, etc.) by their audiences. Representatives of social media in China include WeChat (a social networking application), Weibo (a microblog platform), and TikTok (a short-video application). Chinese audiences may also have had access to overseas media including overseas news websites and social media such as Twitter and Facebook, although the usage of these platforms is relatively scarce.

Geographic location will be classified into three categories according to the degrees of impact of the COVID-19 pandemic. The first is Hubei cities including Wuhan, where the first case of coronavirus in China took place, and which was also the region with the most severe pandemic (49,122 cases in Wuhan and 17,785 cases in other Hubei regions by the end of February). The second was the six cities hit relatively severely by the pandemic, that is, Beijing, Shanghai, Chongqing, Guangzhou of Guangdong Province, Shenzhen of Guangdong Province, and Wenzhou of Zhejiang Province (337–576 cases by the end of February). The third was the other cities that were less impacted by the coronavirus (less than 300 cases by the end of February). These statistics were provided by the National Health Commission of P.R. China as well as the local Health Commissions.

## 2. Materials and Methods

### 2.1. Data Collection

**Figure 3:**
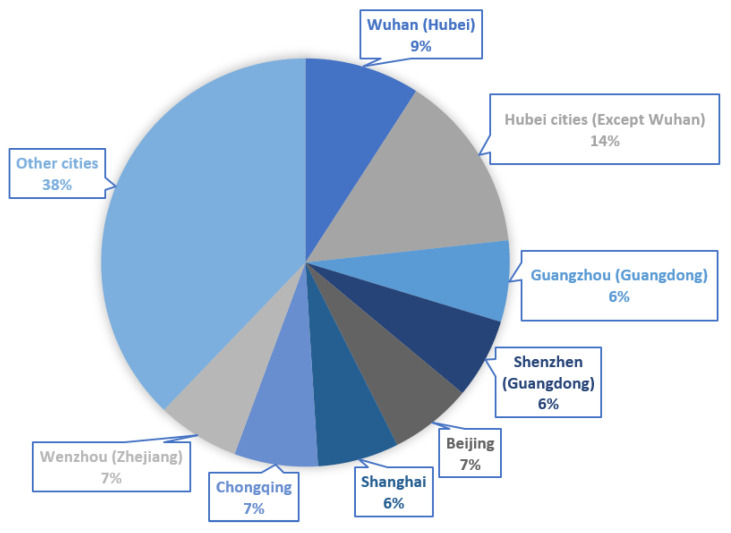
Geographic distribution of the samples.

The data were collected online by a data-collection service provider (i.e., Changsha Ranxing IT Ltd.) during the first two weeks of April 2020 after the most drastic outbreak of coronavirus from late January to February in China. A total of 1118 valid samples were collected, which covered 30 provincial administrative divisions in mainland China. The participation was voluntary and survey responses were anonymous. To ensure that the sample could represent different regions that were impacted by the coronavirus to different degrees, we collected data respectively from the three categories of regions: (1) Hubei cities (23%) including Wuhan (9%), (2) the six cities with relatively severe pandemic including Beijing (7%), Shanghai (6%), Guangzhou (6%), Shenzhen (6%), Chongqing (7%), and Wenzhou (7%), and (3) the other cities that were less impacted by the coronavirus (38%). The regional distribution of the samples is shown in Figure 3. 

### 2.2. Participants

The participants comprised of 45.9% males and 54.1% females. A majority (85.5%) aged between 18 and 40, 4.1% aged below 18, 9.9% aged between 40 and 60, and 0.2% aged above 60. Approximately 80% of the participants had received a university degree. More than half (53.7%) of the participants reported that they were middle class, 36% lower to middle class, and 10.3% middle to upper class. Among the participants, 24% reported an average health condition, 53.7% reported a good health condition, and 20.3% reported an excellent health condition. 

During the coronavirus pandemic from late January to February, 22.5% were staying in Hubei province (4.7% stayed in Wuhan), 36.5% were staying in the six cities with severe pandemic (including Beijing, Shanghai, Chongqing, Guangzhou, Shenzhen, and Wenzhou), and 41.3% were staying in the other cities that were not significantly impacted by the coronavirus. The majority of them were staying with their family (95.1%), 2.6% were staying with their friends/colleagues/classmates/roommates, and 2.3% were staying alone. Detailed demographic characteristics are shown in Table 1.

### 2.3. Measurements

#### 2.3.1. Media Exposure in Terms of Time Length and Media Sources

Participants were asked to recall their media use during the pandemic of COVID-19 from late January to February. Participants reported their time spent on coronavirus information each day and time spent on information irrelevant to coronavirus each day respectively on 5-point scales: (1 = hardly ever, 2 = less than an hour, 3 = 1–3 h, 4 = 3–5 h, 5 = more than 5 h). Participants also reported their use of different media sources including the government official media (e.g., CCTV, People’s Daily, Hubei Daily), commercial media (e.g., The Paper, Sanlian Life Week, Caixin), social media (e.g., WeChat, Weibo, TikTok) and foreign media on 5-point scales (1 = never, 5 = often). More information about this questionnaire is shown in the Appendix A (Table A1).

#### 2.3.2. Media Vicarious Traumatization

The measurement of media vicarious trauma was adapted from Vrklevski and Franklin’s [43] Vicarious Trauma Scale (VTS). The original scale was developed to assess subjective levels of distress associated with working with traumatized clients. The scale was validated among members of the legal profession. This scale was further adapted and applied in the mediated context in this study, and participants were asked to recall the vicarious trauma they experienced in the process of media exposure during the pandemic of COVID-19 from late January to February. It comprised seven items including “I was exposed to distressing news and experiences via media”, “I find myself distressed by reading the stories and situations on media”, and “It is hard to stay positive and optimistic given some of the information I get from the media”. The responses were rated on a 5-point scale (1 = strongly disagree; 5 = strongly agree). The score of this scale ranged from 7 to 35. A higher score indicates a higher level of distress. Cronbach’s alpha of this scale in this study was 0.78. More information about this questionnaire is shown in the Appendix A (Table A1).

#### 2.3.3. Anxiety Status

The measurement of anxiety status was adapted from Zung’s [44] Self-Rating Anxiety Scale (SAS). The scale was developed based on the definition of anxiety, which was defined as a neurosis characterized by anxious overconcern extending to panic and frequently associated with somatic symptoms. The scale was validated among individuals who have clinical anxiety or depressive disorder diagnoses as well as those who do not meet the criteria for a clinical diagnosis in the U.S. Participants of this study were asked to recall their anxiety status during the pandemic of COVID-19 from late January to February. It comprised three questions including “I feel nervous and anxious due to the coronavirus pandemic” and “I have sleeping problems during the coronavirus pandemic”. The responses were rated on a 5-point scale (1 = strongly disagree; 5 = strongly agree). The score of this scale ranged from 3 to 15. The less anxious participant will have a low score on the scale and the more anxious participant will have a higher score. Cronbach’s alpha of this scale in this study was 0.83. More information about this questionnaire is shown in the Appendix A (Table A1).

### 2.4. Statistical Analysis

Data analysis was conducted using SPSS 21.0 statistical software (SPSS Inc., Chicago, IL, USA). The paired-sample t-test was conducted to compare participants’ length of time exposed to coronavirus information and information irrelevant to coronavirus. The Pearson correlation analysis was conducted between anxiety status and the demographic variables (i.e., including age, gender, education, social economic status, and health condition). One-way ANOVAs were conducted to compare the level of anxiety across participants staying in different regions, and with different accommodation statuses. A hierarchical regression was conducted to test the mediation effect of media vicarious traumatization between media exposure and anxiety. Another hierarchical regression was conducted to test the moderation effect of geographic location on the link between media exposure and media vicarious traumatization. The Process Macro (model 7) was conducted to test the moderated mediation model between media exposure and anxiety with media vicarious traumatization as the mediator and geographic location as the moderator.

## 3. Results

### 3.1. Media Exposure and Anxiety Status during COVID-19

#### 3.1.1. Media Exposure

Over 50% of the participants spent 1–3 hours per day on coronavirus information, and 23.4% spent less than one hour. Another 14.8% and 6.5% spent 3–5 hours or more than 5 hours per day. Paired-sample t-test showed that participants spent significantly more time on coronavirus information (M = 3.02, SD = 0.83) than on information irrelevant to coronavirus (M = 2.87, SD = 1.01; t = 3.89, *p* < 0.001). The participants’ frequency of social media use was rated at 4.24 (SD = 0.93), followed by official media use (M = 4.01, SD = 1.10), commercial media use (M = 2.73, SD = 1.15), and overseas media use (M = 1.74, SD = 0.94). 

#### 3.1.2. Anxiety Status

The average level of anxiety for all participants was 2.83 (SD = 0.98). Participants’ anxiety was positively related to age (*r* = 0.11, *p* < 0.01) and negatively related to health condition (*r* = −0.16, *p* < 0.01), but was not significantly related to gender, education, or social economic status. 

The levels of anxiety were significantly different across geographic locations (F = 5.99, *p* < 0.001), and post-hoc analysis showed that participants who stayed in Wuhan during the coronavirus pandemic had a significantly higher level of anxiety (M = 3.36, SD = 0.95) compared to participants who stayed in other Hubei cities (M = 2.75, SD = 1.04; *p* < 0.05), cities with severe pandemic (M = 2.78, SD = 0.99; *p* < 0.05), and other cities (M = 2.85, SD = 0.93; *p* < 0.05).

The levels of anxiety were significantly different across participants who stayed alone, stayed with their family, and stayed with a friend/colleague/classmate/roommate during the coronavirus pandemic (F = 4.04, *p* < 0.05). Post-hoc analysis showed that participants who stayed alone were significantly more anxious (M = 3.36, SD = 1.21) than those who stayed with their family (M = 2.82, SD = 0.97; *p* < 0.05) or with a friend/colleague/classmate/roommate (M = 2.71, SD = 0.99; *p* < 0.05).

### 3.2. Mediation Effect of Media Vicarious Traumatization

The mediation effect of media vicarious traumatization between media exposure and anxiety was tested and results are shown in Table 2. In the first step predicting anxiety, the effects of the use of commercial media (β = 0.11, *p* < 0.01) and overseas media (β = 0.09, *p* < 0.01) were positive and significant after time spent on COVID-19 information was controlled. In the second step predicting media vicarious traumatization, the effects of use of official media (β = 0.06, *p* < 0.05), commercial media (β = 0.11, *p* < 0.001), social media (β = 0.07, *p* < 0.05), and overseas media (β = 0.08, *p* < 0.01) were all positive and significant after time spent on COVID-19 information was controlled. In the third step predicting anxiety with media use variables and media vicarious traumatization, the effect of use of official media was negative and significant (β = −0.05, *p* < 0.05), the effects of use of commercial media (β = 0.04, *p* > 0.05), social media (β = −0.02, *p* > 0.05), and overseas media (β = 0.04, *p* > 0.05) was nonsignificant, and the effect of media vicarious traumatization was positive and significant (β = 0.61, *p* < 0.001).

**Figure 4:**
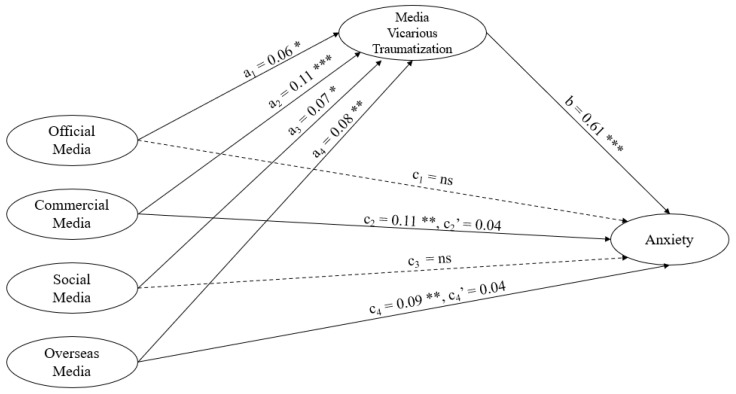
Media exposure and anxiety mediated by media vicarious traumatization. * *p* < 0.05; ** *p* < 0.01; *** *p* < 0.001.

Results showed a full mediation model of media vicarious traumatization between commercial media use and anxiety, and between overseas media use and anxiety. It suggested that commercial media exposure increases anxiety both directly and indirectly by increasing media vicarious traumatization. Similarly, overseas media exposure increases anxiety both directly and indirectly by increasing media vicarious traumatization. In contrast, there is only an indirect effect of official media exposure on anxiety, and an indirect effect of social media exposure on anxiety (See Figure 4, where a denotes the path coefficients from the independent variables to the mediator, b denotes the path coefficient from the mediator to the dependent variable, and c denotes the path coefficients from the independent variables to the dependent variable).

### 3.3. Moderation Effect of Geographic Location

The moderation effect of geographic location on the link between media exposure and media vicarious traumatization was tested. Geographic location (i.e., Hubei cities, cities with severe pandemic, the other cities) was dummy coded (baseline = Hubei cities). In the first block, use of official media, commercial media, social media, overseas media, and dummy variables of geographic location were entered with the time spent on COVID-19 information controlled. In the second block, eight interaction terms formed by the four types of media exposure and the two geographic location dummy variables were entered. Results showed that there was a significant interaction between commercial media exposure and geographic location (i.e., cities with severe pandemic versus Hubei cities; β = 0.25, *p* < 0.05). 

**Figure 5:**
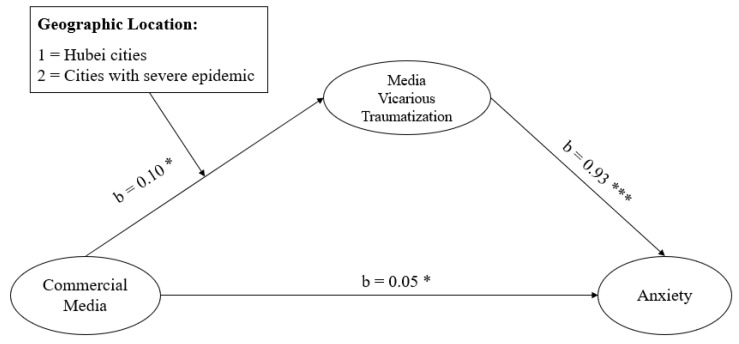
Moderated-mediation model. * *p* < 0.05; *** *p* < 0.001.

The moderated mediation model between commercial media exposure and anxiety with media vicarious traumatization as the mediator and geographic location (1 = Hubei cities; 2 = cities with severe pandemic) as the moderator (as shown Figure 5) was tested with Model 7 of the SPSS PROCESS MACRO (Heinrich-Heine-Universität, Düsseldorf, Germany; Hayes, 2013). All analyses computed a 95% bias-corrected confidence interval (CI) with 5000 bootstrap resamples. Results showed a significant moderated mediation model (b = 0.09, Boot SE = 0.05, 95% Boot CI (0.004, 0.178)). Specifically, the effect of the interaction between geographic location and commercial media exposure on media vicarious traumatization was significant (b = 0.10, *p* < 0.05), the effect of media vicarious traumatization on anxiety was significant (b = 0.93, *p* < 0.001), and the effect of commercial media exposure on anxiety was significant (b = 0.05, *p* < 0.05). The indirect effect of commercial media exposure on anxiety through media vicarious traumatization was significant among those stayed in cities with severe pandemic (b = 0.15, Boot SE = 0.03, 95% Boot CI (0.098, 0.200)), but was not significant among those who stayed in Hubei cities (b = 0.06, Boot SE = 0.04, 95% Boot CI (−0.019, 0.129)). The results suggested that compared to participants who stayed in Hubei, those who stayed in the cities under the severe pandemic were more sensitive to commercial media and were more susceptible to media vicarious traumatization.

## 4. Discussion

This study explored the vicarious traumatization effect caused by the four types of media in the context of crisis events. This study also found that media vicarious traumatization was an important mediator between different types of media exposure and anxiety. There was a full mediation effect of media vicarious traumatization between commercial media exposure and anxiety, as well as between overseas media exposure and anxiety. Meanwhile, there was an indirect-only mediation effect of media vicarious traumatization between official media exposure and anxiety, as well as between social media exposure and anxiety. Geographic location (Hubei cities versus cities with severe pandemic) moderated the effect of commercial media exposure on vicarious traumatization.

### 4.1. Media Vicarious Traumatization

Although not all four media sources cause anxiety directly, all of them cause vicarious traumatization to some extent. Commercial media was the one that was the most strongly linked to vicarious traumatization, followed by overseas media, social media, and official media. More interestingly, it was found that the link between commercial media exposure and media vicarious traumatization was moderated by geographic location, and people staying in cities with severe pandemic, compared to those staying Hubei cities, was more sensitive to commercial media, which relates to higher vicarious traumatization. An explanation for this is that people in Hubei cities experienced the trauma brought by coronavirus directly rather than vicariously from media; they had more reliable and direct information sources, such as their own experiences or the experiences of those people close to them other than media sources. In contrast, people in cities with severe pandemic had much fewer chances to directly experience the trauma events, but had to rely more on media sources, and their perception of the threat of the pandemic was formed by media reports. This was consistent with previous studies showing that geographic location and personal trauma experience may moderate the media effect in causing anxiety in crisis events [39,40,41,42]. This finding also suggests that the effect of vicarious traumatization caused by media exposure will cause more harm to people who are dependent on media and have relatively less direct traumatic experience. When people are directly exposed to higher levels of trauma, the effect of media exposure in causing traumatization vicariously will be undermined. This was in line with the media dependency theory, which claimed that audiences are not reliant on all media equally, and their reliance can be shaped by situational factors [10]. For example, when one perceives a higher ambiguity on the environment and a higher difficulty of understanding the environment in times of crisis, he or she will become especially dependent on mediated information and expert recommendations in order to restore a perceived order to the world [45]. With an increase in the degree of media reliance, the positive or negative influence of media will increase [35]. 

### 4.2. Comparisons Between Commercial Media and Other Media Sources

Through a series of analysis and comparisons in the current study, commercial media was found to cause the highest level of vicarious trauma as well as anxiety compared to official media, social media, and overseas media, and should have particular attention paid to it in the context of crisis events. Characteristics of the influence of commercial media can be summarized as the following: first, it was most strongly associated with media vicarious traumatization compared to other types of media; second, its relation with anxiety was fully mediated by vicarious traumatization; third, its relation with vicarious traumatization was moderated by geographic location. The reason why commercial media causes more trauma and anxiety to audiences can be attributed to the standpoint of the media itself. During the early stages of the COVID-19 pandemic, performances of the commercial media in China were very impressive, and numerous reports became viral on various network platforms. The commercial media, which is market-oriented, usually define themselves as having the role of supervisors and alarms on social issues and anticipate making their own voice different from the grand narrative of the government official media. Therefore, the commercial media need to win the media market with exclusive in-depth reports and must pay more attention to the readability of their content. In this regard, they were more in pursuit of the details of the pandemic and were more inclined to portray micro perspectives. The commercial media also pay more attention to the voice of ordinary people and highlighted the humanitarian issues with regard to the selection of information sources. Therefore, commercial media were more likely to cover ordinary patients and their family members during the outbreak of coronavirus, which conveyed a great amount of anxiety, panic, and even helplessness and despair. These characteristics of commercial media wins more public attention as negative contents are frequently more influential than those of a positive nature [46].

In contrast, as the spokesman of the government, the government official media in China pay more attention to the seriousness, objectivity, authority, and neutrality of information. During the outbreak of the coronavirus, official media reports mostly covered the latest authoritative data and progress of the pandemic, the impact of the pandemic on major areas of society, as well as the official policies and measures. However, the reports of the official media usually lack the micro perspective, the voice of the people, and readability. The self-categorization model of stress suggested that information from an outgroup will result in lower evaluations on threat than information from one’s ingroup [47]. Therefore, if audiences perceive the official media to be psychologically distant and not reflecting their lived experiences, the information presented will be less influential [19].

Effects of social media have been twofold during the pandemic. On the one hand, it has carried massive amounts of information with emotions that were, in most cases, negative. However, on the other hand, it played an important role of social support and emotional catharsis. As was argued by Ball-Rokeach [48], during and following times of crisis, people are dependent on the media not only for information-seeking, but also for tension release or emotional coping purposes. Therefore, the role of social media is made more complex: it exchanges negative emotions rather than merely producing negative emotions. This might be one of the explanations why social media exposure was found not to be directly related to anxiety during the coronavirus pandemic, although it does cause the same vicarious traumatization as other types of media.

Finally, people’s contact and use of overseas media is relatively scarce in China, and the usage of overseas media is relatively high among those with higher education levels. In the case of high uncertainty, some audiences may actively seek information from overseas media for comparison, in order to balance the domestic information sources and to improve the accuracy of their cognition of the objective world. Bias also clearly exists in overseas media. However, it is worth noting that although overseas media was used and relied on the least during the coronavirus pandemic, it can still cause trauma and anxiety, and its negative outcomes should not be ignored. 

### 4.3. Implications

Findings of the current study indicated that vicarious trauma can be formed not only among those who have had direct contact with trauma survivors, but also via repeated media exposure. This is also a noteworthy caution for media practitioners. The media should not only restore the truth and improve readability as much as possible but should also stand in the perspectives of media ethics and humanistic care. They should try their best to avoid either consuming the public affection and privacy of victims or creating secondary trauma for the audiences, especially during a crisis event when the public is already suffering from severe physical and psychological distress.

### 4.4. Limitations

The survey was cross-sectional and was only targeted at the early stage of the COVID-19 pandemic in China. A longitudinal study could be conducted to observe the impact of media in terms of vicarious traumatization at different stages of the pandemic. Future studies should also employ experimental studies to probe which factors in media reports (e.g., framing schemes, microscopic/macroscopic perspectives, emotion expressions, etc.), especially for the commercial media, cause higher levels of trauma and anxiety in their audiences.

## 5. Conclusions

The current study explored the mediation effect of media vicarious traumatization between media exposure and anxiety differences across various media sources, and the moderation effect of geographic location on the link between media exposure and vicarious traumatization. It expanded the concept and application of vicarious traumatization to the mediated context, and the findings provided insightful advice to media practitioners. 

## Figures and Tables

**Table 1:** Demographic Characteristics.

Demographics	Percentage
Sex	
Male	45.9%
Female	54.1%
Age	
Below 18	4.1%
18–25	30.6%
26–30	22.6%
31–35	23.5%
36–40	9%
41–50	7.6%
Above 50	2.5%
Education	
High school or below	12.7%
College/university	79.8%
Postgraduate	7.5%
Social Economic Status	
Lower to middle class	36.0%
Middle class	53.7%
Middle to upper class	10.3%
Health Condition (Mean = 3.92, SD = 0.72)	
Very poor	0.1%
Relatively poor	2.0%
Average	24.0%
Relatively good	53.7%
Very good	20.3%
Location of Residence During the Pandemic	
Hubei (Wuhan)	4.7%
Hubei (excluding Wuhan)	17.8%
Cities with severe pandemic (including Beijing, Shanghai, Chongqing, Guangzhou in Guangdong province, Shenzhen in Guangdong province, and Wenzhou in Zhejiang province)	36.2%
Other cities (not significantly impacted by the coronavirus)	41.3%
Accommodation	
Staying with family	95.1%
Staying with friends	2.6%
Staying alone	2.3%

SD: Standard Deviation.

**Table 2:** Testing the mediation effect of media vicarious traumatization between media exposure and anxiety.

Variables	Outcome: Anxiety	Outcome: Media Vicarious Traumatization	Outcome: Anxiety
Time Spent on COVID-19 Information	0.19	0.16	0.09 ***
Official Media Use	−0.01	0.06 *	−0.05 *
Commercial Media Use	0.11 **	0.11 ***	0.04
Social Media Use	0.02	0.07 *	−0.02
Overseas Media Use	0.09 **	0.08 **	0.04
Media Vicarious Traumatization			0.61 ***

* *p* < 0.05; ** *p* < 0.01; *** *p* < 0.001.

## Appendix A

**Table A1:** Questionnaire List.

Questionnaires	Indicators
Demographics	
Sex	1 = male 2 = female
Age	1 = below 18 2 = 18–25 3 = 26–30 4 = 31–35 5 = 36–40 6 = 41–50 7 = above 50
Education	1 = primary school 2 = middle school 3 = high school 4 = college/university 5 = postgraduate
Social Economic Status	1 = lower class 2 = lower to middle class 3 = middle class 4 = middle to upper class 5 = upper class
Health Condition	1 = very poor 2 = relatively poor 3 = average 4 = relatively good 5 = very good
Location	1 = Hubei (Wuhan) 2 = Hubei (excluding Wuhan) 3 = cities with severe pandemic (including Beijing, Shanghai, Chongqing, Guangzhou, Shenzhen, and Wenzhou) 4 = other cities
Accommodation	1 = staying with family 2 = staying with friends/colleagues/classmates/roommates 3 = staying alone
**Media Exposure**	
1. Time spent on coronavirus information each day	1 = hardly ever 2 = less than an hour 3 = 1–3 h 4 = 3–5 h 5 = more than 5 h
2. Time spent on information irrelevant to coronavirus each day	1 = hardly ever 2 = less than an hour 3 = 1–3 h 4 = 3–5 h 5 = more than 5 h
3. Use of different media sources—official media (e.g., CCTV, People’s Daily, Hubei Daily)	1 = never 5 = often
4. Use of different media sources—commercial media (e.g., The Paper, Sanlian Life Week, Caixin)	1 = never 5 = often
5. Use of different media sources—social media (e.g., WeChat, Weibo, TikTok)	1 = never 5 = often
6. Use of different media sources—overseas media	1 = never 5 = often
**Media Vicarious Traumatization**	
1. I was exposed to distressing news and experiences via media.	1 = strongly disagree 5 = strongly agree
2. I find myself distressed by reading the stories and situations on media.	1 = strongly disagree 5 = strongly agree
3. It is hard to stay positive and optimistic given some of the information I get from the media.	1 = strongly disagree 5 = strongly agree
4. I find myself thinking about distressing news on media.	1 = strongly disagree 5 = strongly agree
5. Sometimes I feel helpless because I cannot give help to people in need.	1 = strongly disagree 5 = strongly agree
6. Sometimes I feel overwhelmed by reading the media reports.	1 = strongly disagree 5 = strongly agree
7. I find it difficult to deal with the media content.	1 = strongly disagree 5 = strongly agree
**Anxiety Status**	
1. I feel nervous and anxious due to the coronavirus pandemic.	1 = strongly disagree 5 = strongly agree
2. I have sleeping problems during the coronavirus pandemic.	1 = strongly disagree 5 = strongly agree
3. I feel panicky and cannot sit still easily during the coronavirus pandemic.	1 = strongly disagree 5 = strongly agree
